# Biaxial 360-degree scanning LIDAR using a liquid crystal control

**DOI:** 10.1038/s41598-021-94208-2

**Published:** 2021-07-20

**Authors:** Seiji Nishiwaki

**Affiliations:** Technology Division, Panasonic Corporation, 3-1-1 Yagumo-nakamachi, Moriguchi City, Osaka 570 - 8501 Japan

**Keywords:** Engineering, Optics and photonics

## Abstract

Sophisticated non-mechanical technology for LIDARs is needed to realize safe autonomous cars. We have confirmed the operating principle of a non-mechanical LIDAR by combining concentric circular-grating couplers (CGCs) with a coaxially aligned rod lens. Laser light incident vertically on the center of the inner CGC along the center axis of the lens is radiated from the outer CGC and passes through the side surface of the lens. It is converted to a parallel beam that scans in two axes by applying voltages to two area-segmented electrode layers sandwiching the CGCs and a liquid crystal layer formed on the CGCs. We have demonstrated scanning whose motion ranges were 360 degrees horizontally and 10° vertically. A beam with a spread angle of 0.3° × 0.8° at a minimum swept vertically up to a frequency of 100 Hz and ten equally spaced beams scanned rotationally with a 6-degree cycle variation of spread of between 0.8° and 3.5°.

## Introduction

Aiming for completion in the next few years, auto manufacturers and information equipment manufacturers across the globe are advancing the development of fully autonomous cars. To realize this “level 5,” real-time measurement technology of distance between cars and physical objects is necessary. LIDAR (Light detection and ranging) apparatus using time of flight (TOF) technology is one potential solution. Conventional LIDARs are composed of a mechanical structure^1–3^ such as a polygon mirror or a galvanic mirror mounted on a rotating plate. The downside of this type is that they are expensive, slow, large, heavy, and non-durable due to having moving parts. To overcome these drawbacks, various non-mechanical methods (e.g., solid state LIDAR or SS-LIDAR) have been proposed.

A MEMS (microelectromechanical system) mirror^4–7^ or an optical phased array^8–16^ (OPA) can be used for SS-LIDAR. A MEMS mirror is fabricated by etching a silicon crystal and forming a coil structure. The direction of a light ray incident on the mirror surface can be controlled by the Lorentz force produced by energization of the coil. MEMS-LIDAR is likely the most advanced non-mechanical type, but it faces problems with durability because it still has moving parts. Moreover, it is difficult to steer it free from hysteresis caused by creep and it is also easily affected by automotive vibrations due to the use of a resonant mode (non-linear mode) for scanning in the horizontal direction.

For the OPA, although phased arrays are practically realized in the field of radio wave technology, manufacturing challenges remain with the fine-pitched fabrication process that is required. It is also difficult to control the phases emitted from each array and to correct for environmental changes during use.

Another proposition provides a good light sweeper by using a slow-light waveguide^17–19^, in which light is transported with the slow group velocity in multiple layers or photonics crystals. However, few slow-light methods appear to be suitable for commercial use because their beam steering that exploits wavelength changes requires an expensive light source.

Some electro-optic structures^20,21^ and photonic-crystal lasers^22,23^ give provide a good light scanner, but they are limited to one-dimensional steering.

All the above-mentioned SS-LIDARs significantly narrow their steering ranges to realize non-mechanical scanning and most of them (except for MEMS-LIDARs) need detection structures that are physically separate from their emission structures. To provide a robust answer to these challenges, we devised a novel structure (named CGC-LIDAR) using an LC (liquid crystal) and demonstrated the light-scanning principle over a 360-degree sweeping range.

### Structure and operational principle of a CGC-LIDAR

**Figure 1:**
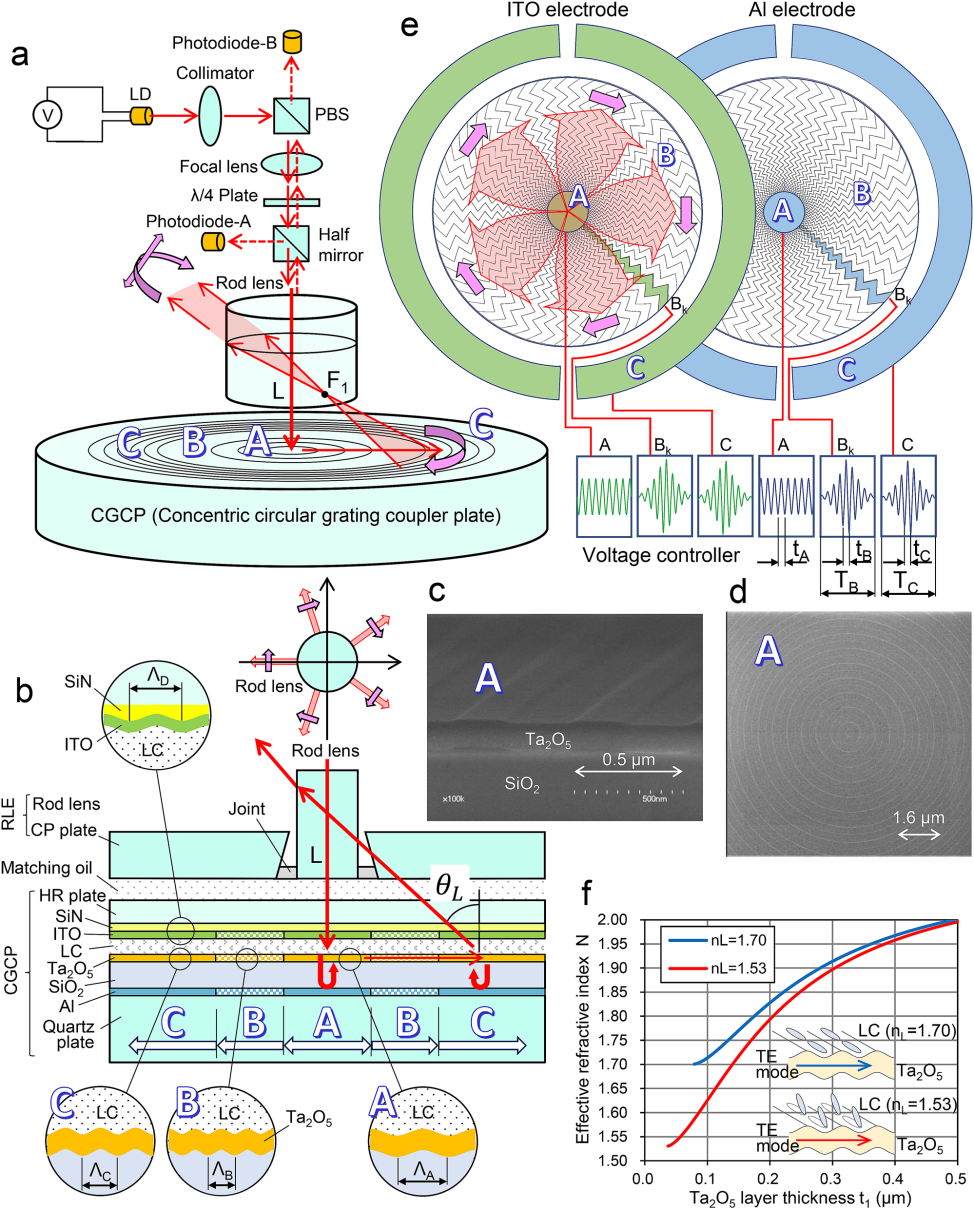
Structure and operational principles of CGC-LIDAR. (**a**) Perspective illustration of a CGC-LIDAR. The circularly polarized light vertically incident to coupler A excites a guided light which propagates uniformly from the center to outside. The guided light is radiated from coupler C, concentrated near point F_1_, and converted to 2D-scannable five parallel beams by refraction at the side surfaces of the rod lens. (**b**) Cross-sectional illustration of a CGC-LIDAR. Concentric circular gratings are formed on the Ta_2_O_5_ / SiO_2_ layers of the quartz plate and on the ITO/SiN layers of the HR plate. The upper and lower gratings and electrodes are divided from the inside to outside into the three areas of A, B, and C. The LC layer sandwiched by the two plates is homogeneously aligned along the rotational direction around the axis L due to the orientation force of the gratings. CGC-LIDAR is built up concentrically with an RLE and a CGCP which are joined with a matching oil. (**c**) Perspective-view SEM photographs of coupler A. (**d**) Top-view SEM photograph of coupler A. (**e**) Configuration diagram of electrodes for areas A, B, and C. The electrode B is divided into 60 areas of B_k_ (k = 0 to 59) in the rotational direction. While the electrodes A and C, shaped as circles or half rings, have the same shapes between the Al layer and the ITO layer, the B_k_ electrodes with a zigzag shape are reversed between them. The electrode C is divided in half and the gaps between them are used for the wires of electrodes A and B_k_. Since the AC signals applied to the electrodes are reversed between the Al electrode and the ITO electrode, the differences in voltages between them are doubled. (**f**) Relationships between effective refractive index (ERI) and waveguide thickness. When AC signals are applied to the electrodes, the LC alignment is raised, and ERI falls in inverse proportion to the AC amplitude.

Figure 1a,b are a perspective and cross-sectional illustration of a CGC-LIDAR that includes concentric grating couplers (CGCs)^24–26^. Light of wavelength λ = 0.94 µm emitted from a laser diode is collimated to parallel light by a collimator and is reflected to a polarizing beam splitter (PBS). It is then focused by a focal lens on the inner CGC, passing through a 1/4 wave plate, a half mirror, and the rod lens along axis L. The circularly polarized light vertically incident on the CGC excites the guided light in TE mode in the waveguide layer which propagates uniformly from the center to outside. The guided light is radiated from the coupler C and is concentrated near point F_1_ by LC control. It is then converted to a parallel beam by refraction at the side surfaces of the rod lens. Five parallel beams are generated equiangularly around the rod lens and are rotated by LC control. Diffused light reflected from an outer object returns to the coupler C and excites the guided light in the opposite direction. The return light output from the coupler A is detected by photodiodes A and B. TOF signals are produced by the two detectors. Our method produces multi-sweep beams in principle, and these beams also produce individual TOF signals. They can be separated by using the two detectors (see Supplementary information S1).

CGC-LIDAR is built up concentrically with an RLE (rod lens element) and a CGCP (CGC plate) which are joined with a matching oil of refractive index 1.59. The RLE consists of a rod lens (radius r_0_ = 0.95 mm) and a CP (circularly perforated) plate, which are built along axis L and fastened together with a joint that aligns their bottom surfaces (see Supplementary information S2). The CGCP is built of a quartz plate and an HR (high refractive index, index 2.0) plate by encapsulating a 5 µm-thick LC layer, made of 5CB.

On the LC-side surface of the quartz plate, an aluminum (Al) layer (0.09 µm thick), a SiO_2_ (silicon oxide) layer (t_0_ = 1.16 µm thick) and a Ta_2_O_5_ (tantalum oxide) layer (t_1_ = 0.132 µm thick) are formed sequentially. The Al layer acts as an electrode (or a reflector) and is divided from inside to outside into three areas: A, B, and C (radius 0–0.10 mm, 0.30–4.01 mm, and 4.50–7.00 mm).

As shown in Fig. 1c,d, concentric circular gratings (d = 0.013 µm deep) divided into three areas of A, B, and C (radius 0–0.05 mm, 0.05–4.50 mm, and 4.50–6.50 mm) are formed concentrically along the axis L by electron lithography on the SiO_2_ layer after a planarizing process using CMP (chemical mechanical polishing). The SiO_2_ layer acts as a buffer layer and the Ta_2_O_5_ layer acts as a waveguide. While the gratings in areas A and C behave as grating couplers because their grating pitches (Λ_A_ = 0.544 and Λ_C_ = 0.295 µm) are larger than the critical level of coupling (0.27 µm), the grating area B does not behave as a coupler because its pitch (Λ_B_ = 0.240 µm) is smaller. The duty rates ε of the gratings in area A and B are 0.5, and that in C is 0.2 (see Supplementary information S3).

Whereas, a SiN (silicon nitride) layer (0.10 µm thick) and an ITO (indium tin oxide) layer (0.10 µm thick) are formed on the LC-side surface of the HR plate. Concentric circular gratings (0.05 µm deep, period Λ_D_ = 1.2 µm) are concentrically formed from inside to outside by reduced projection exposure along the axis L on the surface of the SiN layer. The ITO layer behaves as an electrode, and in the same way as the Al layer, is divided into three areas: A, B, and C.

As shown in Fig. 1e, the electrode B is further divided into 60 areas of B_k_ (k = 0 to 59) in the rotational direction. The electrode C is divided into halves to lace wires to the outside. While electrodes A and C have the same shapes between the Al layer and the ITO layer, the B_k_ electrodes, which have a zigzag shape (see Supplementary information S4), are reversed between them. Although the B_k_ electrodes do not need wiring because their shapes are equivalent to wiring, they must use the spaces between electrodes B and C (i.e., radius 4.01–4.50 mm) to link them to the outside. For electrode A, one electrode of B_k_ is used as a wire instead. Rectangular or triangular AC (alternating current) voltages of cycle t_A_, t_B_, and t_C_, respectively, are applied to electrodes A, B, and C. While an envelope waveform of constant AC amplitude is applied to electrode A, varying waveforms are applied to the electrodes of B_k_ and C with cycle T_B_ and T_C_, which are related to beam sweeping cycles in the horizontal and vertical directions. Since the AC signals are reversed between the Al electrode and the ITO electrode, the differences in voltages between them are doubled.

There is no other alignment structure (or surface treatment) than the gratings. Thus, the LC layer sandwiched by the two plates is homogeneously and concentrically aligned in the rotational direction around the axis L due to the orientation force of the gratings remaining on the surfaces of the Ta_2_O_5_ layer and the ITO layer (see Supplementary information S5). As shown in Fig. 1f, when AC signals are applied to the electrodes, the alignment of the LC molecules is raised in a plane contacting both the thickness direction of the LC and the grating direction. The effective refractive index (ERI) of N for the guided light declines as a function of increasing AC amplitude due to change of n_L_ (i.e., the refractive index of the LC layer for the guided light of TE mode).

The voltage control of electrode A optimizes the coupling conditions at the coupler A. The voltage of electrode C controls the vertical scan according to the formula of

1 $$ n_{L} \sin \theta_{L} = \lambda /{{\Lambda }}_{C} - N, $$

where θ_L_ is the radiation angle in the LC layer (see Supplementary information S2). The waveforms applied to the B_k_ electrodes adjust the focusing position F_1_ to convert the radiated light to five parallel beams (see Supplementary information S6). Rotation of this waveform produces a horizontal scan around the axis L.

### Beam-collimating and rotating principle in the horizontal direction

**Figure 2:**
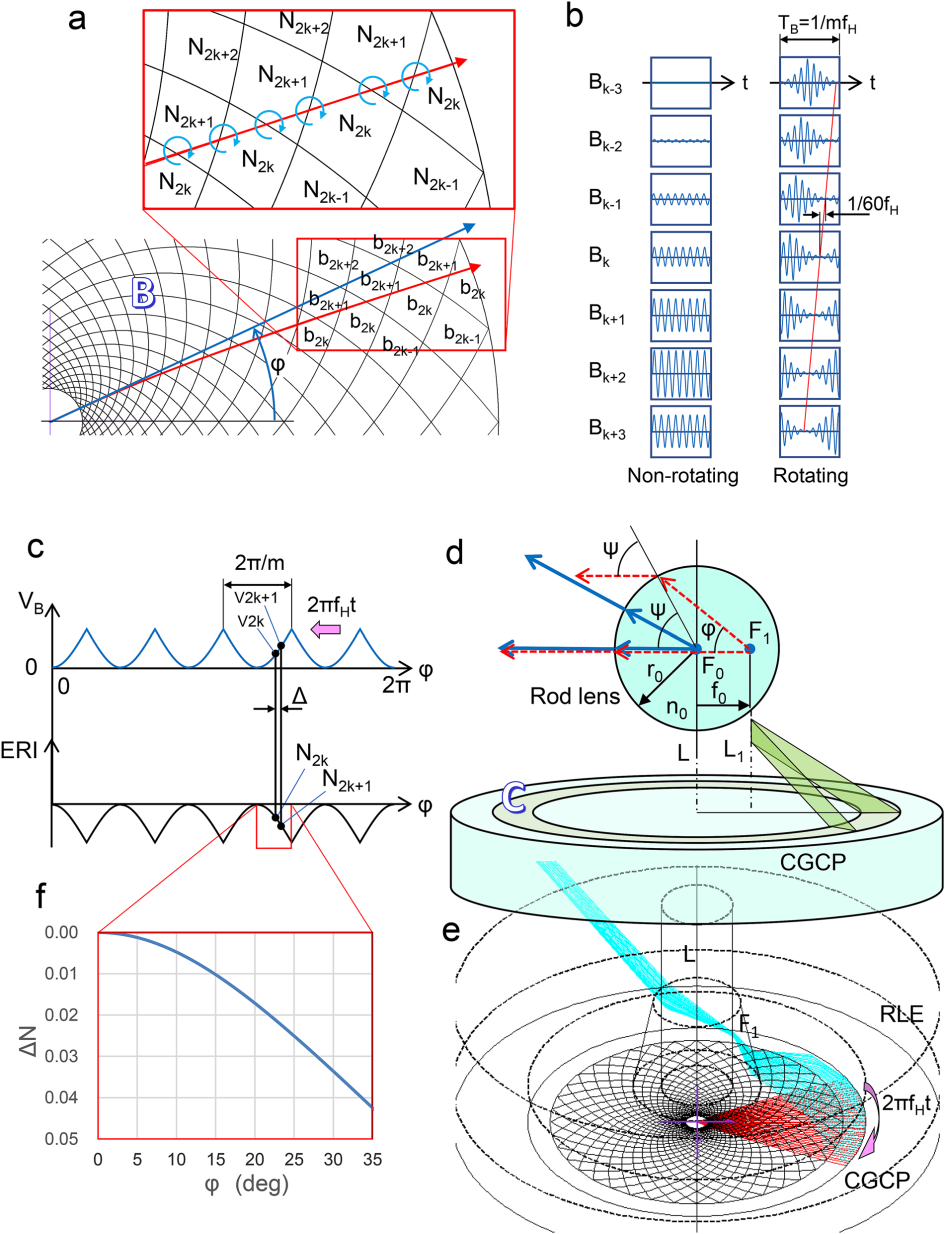
Beam-condensing and rotating principle in the horizontal direction. (**a**) Electrode pairs composed of upper and lower electrodes B_k_ and a trace of guided light. Electrodes b_2k_ are generated from the combination of Al electrodes B_k_ and ITO electrodes B_k_, and electrodes b_2k+1_ are from Al electrodes B_k_ and ITO electrodes B_k+1_. In response to the voltages v_2k_ or v_2k+1_ at electrodes b_2k_ or b_2k+1_, the ERIs of N_2k_ or N_2k+1_ are aligned in the radial direction. The guided light deflects to the higher-index side at the intersection with the boundary of the diamond-shaped electrodes, as shown by the red arrow. (**b**) Examples of voltage waveforms applied to the B_k_ electrodes. Amplitude distribution between the electrodes B_k_ allows the beams to be collimated. Collimated beams can be rotated by applying a phase-shift signal to the neighbor electrodes. **c.** Relationships under a static condition between a voltage waveform and the response of the ERI to an azimuth angle φ. The waveform is represented by a collection of voltage elements v_2k_ and v_2k+1_ which are applied to the electrodes b_2k_ and b_2k+1_. The curve of ERI traces a shape that is the inverse of waveform V_B_. (**d**) Perspective illustration of light radiated from the coupler C and a cross-sectional drawing of light refracted at the rod lens surface. When applied to the B_k_ electrodes, the radiated light is focused on point F_1_ and becomes a parallel beam after refraction at the side surface of the rod lens. (**e**) Ray-traced result for light that propagates in the guided layer and penetrates the RLE. This is an example for m = 5. In the range of φ = –36 to 36 degrees, the guide light is convergent and radiates from the coupler C. It becomes a parallel beam after focusing at point F_1_ and passing through the rod lens. This situation is similar in the other angular ranges and five parallel beams are produced in total. (**f**) Necessary shape of the curve of the ERI difference.

Figure 2a shows a portion of an electrode pair composed of the ITO and Al electrode, in which vertexes of zigzag shapes between the two electrodes of B_k_ overlap together and diamond shape electrodes such as b_2k_ and b_2k+1_ are aligned in the radial direction, where b_2k_ are generated from the combination of Al electrodes B_k_ and ITO electrodes B_k_, and b_2k+1_ are from Al electrodes B_k_ and ITO electrodes B_k+1_. The voltage amplitude at the electrodes b_2k+1_ becomes the average between those at the electrodes b_2k_ and the electrodes b_2k+2_. When the AC signals are applied to the electrodes of B_k_, the ERIs of such as N_2k_ or N_2k+1_, in response to the voltages v_2k_ or v_2k+1_ at electrodes b_2k_ or b_2k+1_, are aligned in the radial direction.

Figure 2b show examples of voltage waveforms applied to the B_k_ electrodes. For a non-rotating condition (or a static state), the AC waveform envelopes are constant, with their signal amplitudes varying sinusoidally according to the value of k. Amplitude distribution between the electrodes allows the beams to be collimated. Under rotating conditions, the envelopes have a sinusoidal shape and the phase between the neighbor signals shifts together by 1/60f_H_. Collimated beams can be rotated by applying a phase shift to the sinusoidal amplitude.

Figure 2c shows relationships under a static condition between a voltage waveform V_B_ applied to the B_k_ electrodes and the response of the ERI to an azimuth angle φ (= 2kπ/60 or (2 k + 1) π/60). The waveform V_B_ has a periodic number m (m = 5 or 10) per revolution and is represented by a collection of elements of v_2k_ or v_2k+1_. N_2k_ (or N_2k+1_) is given from v_2k_ (or v_2k+1_) and its curve traces a shape that is the inverse of waveform V_B_. If N_2k_ > N_2k+1_ as shown in Fig. 2c, the guided light (shown by the red arrow in Fig. 2a) deflects to the higher index side (in the direction of the blue circling arrows) at the intersection with the boundary of the diamond-shaped electrodes. The differences in indexes are small. However, since the guided light passes through more than 30 boundaries within the range of area B, the accumulated deflection angle of the red arrow eventually totals about 10 degrees in comparison with the blue and straight arrow. This deflecting principle is similar to that of an electro-optic scanner^20^. The rotating condition corresponds to the movement of the waveform at a frequency f_H_ as indicated by an arrow shown in Fig. 2c (see information S7).

Figure 2d shows a combination of a perspective illustration of light radiated from the coupler C and a cross-sectional drawing of light refracted at the rod lens surface. Figure 2e shows a ray-tracing result for light that propagates in the guided layer, is then radiated from the coupler C, and penetrates RLE. If no difference in voltage is applied to the B_k_ electrodes, the guided light propagates in the direction of the blue arrow in Fig. 2a: the radiated light is focused at point F_0_ on the center axis L of the rod lens and travels in a straight line in the radial direction as shown by the blue arrows in Fig. 2d. In this case, the radiated light becomes a widely spread beam with a conical wavefront. When applied to the B_k_ electrodes, the guided light propagates in the direction of the red arrow in Fig. 2a and the radiated light is focused on point F_1_, which moves the distance of f_0_ from F_0_. The light becomes a parallel beam after refraction at the side surface of the rod lens, as shown by the red dotted arrows. This beam is generated by the period number m of the waveform, that is, five or ten. To correct the aberration of the radiated beams (see Supplementary information S8), the light focused on F_1_ needs a phase difference of $n_{0 } f_{0} (1 - cos\varphi),$where

$$ f_{0} = \frac{{r_{0 } \sin \psi }}{{n_{0} \sin \varphi }} , \quad \sin \psi = \frac{{n_{0} \sin \varphi \sqrt {n_{0}^{2} + 1 + 2n_{0} \cos \varphi } }}{{\sqrt {\left( {n_{0}^{2} - 1} \right)^{2} + 4n_{0}^{2} \sin^{2} \varphi } }} . $$

Therefore, the difference in ERI caused in the range of the area B (radius r_1_ to r_2_) becomes ${\Delta N} = \frac{{n_{0 } f_{0} \left( {1 - cos\varphi } \right)}}{{r_{2} - r_{1} }}$, as shown in Fig. 2f.

The focal line L_1_ generated by the aggregate of point F_1_ is not actually parallel with the axis L, and slightly lists so as to broaden towards the top with aberrations produced in the horizontal direction. Therefore, to correct those aberrations, an inverted truncated cone is better than a rod shape (see Supplementary information S2).

### Analytical results for coupling characteristics and design condition

Coupler A should ideally be shorter because its coupling efficiency becomes insensitive to changes in wavelength and incident angle. Coupler C needs to be longer because the spread angle of the radiated beam is inversely proportional to the beam width (i.e., coupling length). It is necessary to satisfy these two contradictory conditions by adjusting the coupling length L and the groove duty rate ε, with the grating depths d of couplers A and C remaining the same because they use the same etching processes.

**Figure 3:**
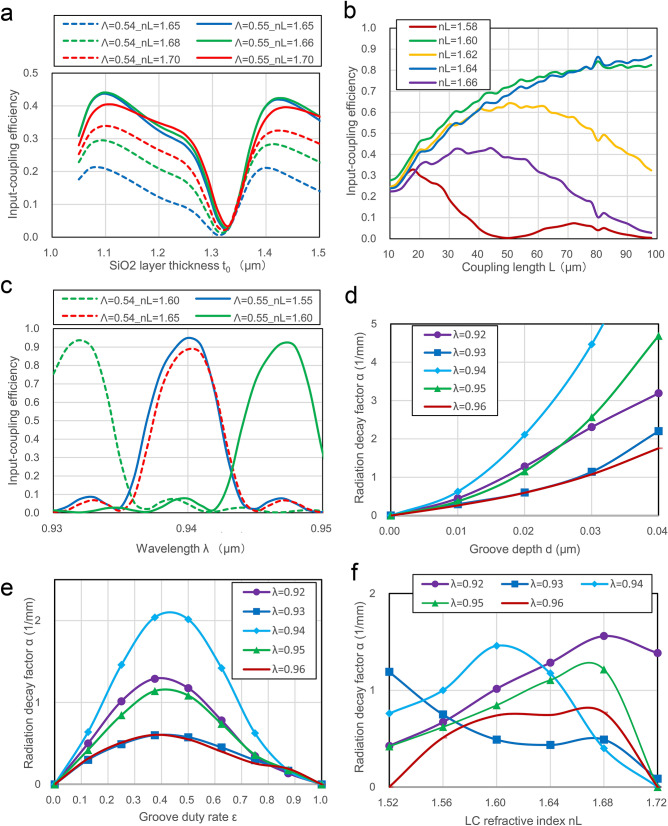
Analytical results for input- and output-coupling characteristics. (**a**) Response of input-coupling efficiency at coupler A to a buffer layer thickness t_0_, where a circularly polarized Gaussian light is vertically incident. This is calculated by 3D-FDTD for λ = 0.94 µm, Λ = 0.54 and 0.55 µm, t_1_ = 0.14 µm, groove depth d = 0.1 µm, groove duty rate ε = 0.5, and diameter L = 10 µm: t_0_ = 1.16 µm is appropriate for counteracting film thickness error. (**b**) Responses of input-coupling efficiency at coupler A to coupling length L where an S-polarized light is vertically incident. This is calculated by 2D-FDTD for λ = 0.94 µm, Λ = 0.55 µm, t_1_ = 0.14 µm, t_0_ = 1.16 µm, d = 0.02 µm, and ε = 0.5. (**c**) Responses of input-coupling efficiency at coupler A to wavelength λ where an S-polarized light is vertically incident. This is calculated by 2D-FDTD for Λ = 0.54 and 0.55 µm, d = 0.02 µm, ε = 0.5, t_1_ = 0.14 µm, t_0_ = 1.16 µm, and coupling length L = 90 µm. (**d**–**f**) Responses of radiation decay factor α for TE mode light at coupler C to groove depth d (where ε = 0.5 and n_L_ = 1.60), groove duty rate ε (where d = 0.02 µm and n_L_ = 1.60), and LC index n_L_ (where d = 0.02 µm and ε = 0.25). These are calculated by 2D-FDTD for Λ = 0.30 µm, t_1_ = 0.14 µm, and t_0_ = 1.16 µm: the factor α can be brought below 1 mm^–1^ by adopting the design condition of d = 0.02 µm, and ε = 0.2.

Figure 3a–c show the responses of input-coupling efficiency at coupler A to the buffer layer thickness t_0_, coupling length L, and wavelength λ, respectively, with the parameters of the refractive index n_L_ of the LC layer, where light is vertically incident. Since 3D-FDTD calculations over side 10 µm far exceed available computing capacity, Fig. 3b,c are analyzed in the 2D model where incident light is S-polarized and uniformly distributed, while maintaining the other structural dimensions. Figure 3a is designed to show to what extent a circular polarized Gaussian beam can be input-coupled to the coupler A in the 3D model while decreasing coupling size, but instead by increasing the groove depth to accurately maintain the input-coupling efficiency.

Figure 3a is calculated under the condition of λ = 0.94 µm, Λ = 0.54 and 0.55 µm, t_1_ = 0.14 µm, d = 0.1 µm, ε = 0.5, and diameter L = 10 µm. Figure 3b,c result for λ = 0.94 µm, Λ = 0.55 µm, t_1_ = 0.14 µm, t_0_ = 1.16 µm, d = 0.02 µm, and ε = 0.5, and for Λ = 0.54 and 0.55 µm, d = 0.02 µm, ε = 0.5, t_1_ = 0.14 µm, t_0_ = 1.16 µm, and coupling length L = 90 µm, respectively.

From Fig. 3a, a coupling efficiency exceeding 40% is anticipated by controlling the thickness t_0_, and t_0_ = 1.16 µm appears to be appropriate for counteracting film thickness error. If the coupling length or grating depth of coupler C is sufficient, all the input-coupled light at coupler A is radiated from C and is converted to collimated beams.

From Fig. 3b,c, by controlling the LC index n_L_, sufficient efficiency for coupler A can be achieved by the design condition of d = 0.02 µm and ε = 0.5, and L = 90–100 µm and Λ = 0.54–0.55 µm are appropriate for maximizing the coupling efficiency.

Figure 3d–f show responses of a radiation decay factor α for TE mode light at coupler C to groove depth d (where ε = 0.5 and n_L_ = 1.60), groove duty rate ε (where d = 0.02 µm and n_L_ = 1.60), and LC index n_L_ (where d = 0.02 µm and ε = 0.25), respectively, with parameters of a wavelength λ, calculated by 2D-FDTD for Λ = 0.30 µm, t_1_ = 0.14 µm, and t_0_ = 1.16 µm. When guided light propagates along a leaky waveguide such as a grating coupler, its light amplitude along the x axis decays exponentially as a function of exp(− αx). This coefficient α is defined as the radiation decay factor, which increases with increased d and as ε approaches 0.5^27^. The value of 1/2α corresponds to the effective coupling length.

From Fig. 3d–f, to increase the coupling length of coupler C, the factor α can be less than 1 mm^−1^ (that is, the radiation range can exceed 0.5 mm) under the design conditions of λ = 0.93–0.94 µm, d = 0.02 µm, and ε = 0.2.

Based on the above results, coupler A is designed under the conditions of Λ = 0.544 µm (tuned by taking the wavelength error into account), d = 0.02 µm (finally fabricated at 0.013 µm), ε = 0.5 and radius 50 µm, and coupler C is designed under the conditions of Λ = 0.295 µm, ε = 0.2 and L = 1.5 mm.

## Experimental system

**Figure 4:**
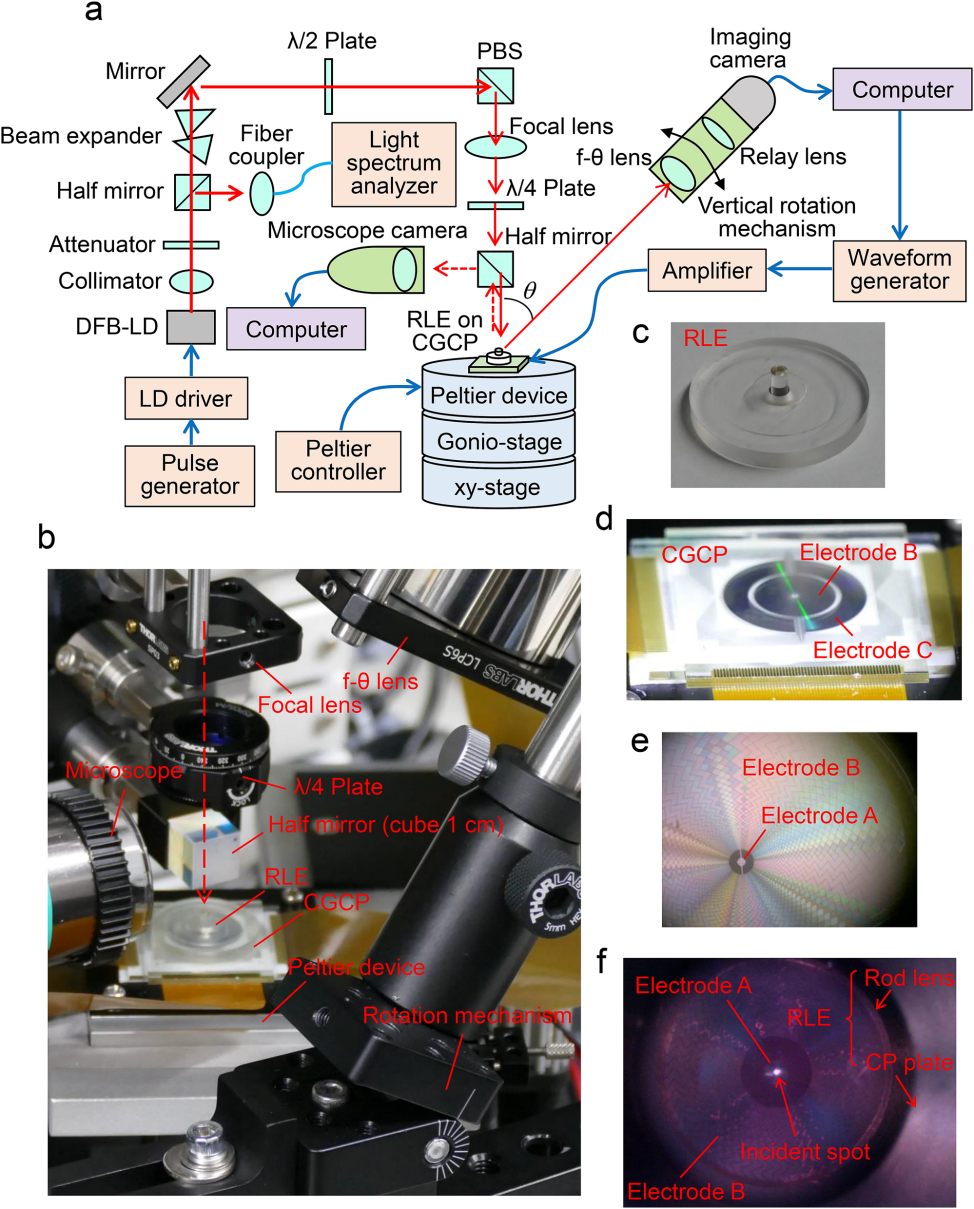
Experimental system for observation of scanning beams. (**a**) Configuration diagram of the experimental system. Light radiated from coupler C is converted from rotation at a constant angular velocity to a linear uniform motion by an f-θ lens and is observed using an imaging camera. (**b**–**d**) Perspective photographs of a portion of the experimental system, the RLE, and the CGCP. (**e**) Photograph for a central surface of the CGCP under LC control, with RLE removed. (**f**) Observation photograph of a light spot incident to the surface of the CGCP using a microscope.

We can observe a scanning beam radiated from the CGCP sample using the experimental system. Figure 4a shows a configuration diagram of the system (see Methods in detail). Figure 4b–d show perspective photographs of a portion of the system, the RLE, and the CGCP. Figure 4e,f, respectively, are micrographs of the surface of the CGCP where the RLE is removed and located.

Light emitted from a DFB-LD (distributed-feedback laser diode, λ = 0.936 µm measured) is collimated and vertically focused to the CGCP with circular polarization. Since a portion of the light incident to the CGCP is reflected from coupler A and the half mirror, the focused spot is observed with the RLE and the CGCP by a microscope camera. Light radiated from coupler C is converted from rotation at a constant angular velocity to a linear uniform motion using an f-θ lens. It is collimated by a relay lens and observed by an imaging camera. Control signals applied to the electrodes of the CGCP are produced in computer-controlled waveform generators and are magnified by an amplifier. Figure 4e is a photograph of a central surface of the CGCP under LC control, with the RLE removed. Since the refractive indexes of the LC layer change under LC control, the external appearance is rotated according to the rotation of the diamond shapes in the radial direction (see Supplementary movie M1). Figure 4f shows an observation photograph of a light spot incident to the surface of the CGCP using a microscope. When the spot is adjusted at the center of coupler A, the radiation from coupler C is confirmed by the imaging camera attached to the f–θ lens.

## Experimental results

Generally, although conventional LC devices including displays are not affected by the frequency of AC voltage, our device is affected. This is because while conventional devices exploit the change in alignment of the LC molecules across the whole layer thickness, our device exploits the change near the boundary face (i.e., in the evanescent field of the guided light). While the LC molecules tilt at an angle according to the electric field intensity, they continuously ramp, near the boundary face, from angle zero to the angle mentioned above (see Supplementary information S5). This boundary molecules are strongly affected by the frequency of AC voltage.

The voltage V_A_ for electrode A was set here to zero, because the amount of light radiated from coupler C reached its maximum for V_A_ = 0. This departure from the adjustable range may be due to a thickness error of t_1_ (i.e., 0.140 µm designed vs. 0.132 µm measured).

**Figure 5:**
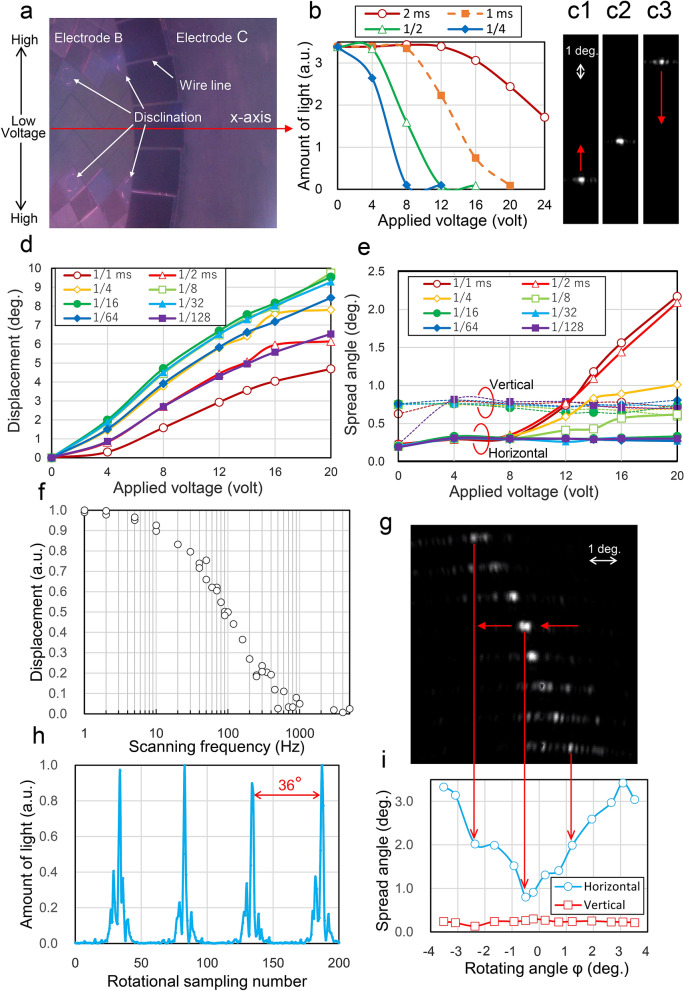
Observation results. (**a**) Observation photograph indicating oriented states of the LC at the fringe region of the electrodes B and C. Electrode B is subject to a static state of m = 10 and V_B_ = 3.2–40.0 V with t_B_ = 1.0 ms. (**b**) Relationship between the amount of light radiated from the coupler C and the constant voltage applied to the electrodes B_k_ with the cycle t_B_ of triangular AC voltage as the parameter. (**c1**–**c3**) Observation photographs of vertically sweeping beams radiated from coupler C using the imaging camera of the f-θ lens. The beams sweep by a vibrating control of T_C_ = 1.0 s and V_C_ = 0–20.0 V for electrode C. (**d**, **e**) Displacement and spread angles of vertical steering beams for the voltage applied to the electrode C with the cycle t_C_ of rectangular AC voltage as a parameter. The Peltier device is set at 26 °C. (**f**) Frequency response of the vertical displacement. Half maximum of the displacement amplitude is maintained up to 100 Hz. (**g**) Observation photograph of rotationally sweeping beams radiated from coupler C using the imaging camera of the f-θ lens. The beams show rotational sweeps by the rotational state of T_B_ = 0.5 s for electrode B and the static state of t_C_ = 1/16 ms and V_C_ = 15.0 V for electrode C. (**h**) Image-analyzed results of a movie, showing amount of light. The beams sweep rotationally 10 times per turn. (**i**) Relationships between the spread angle and the rotating angle. Beams scan rotationally with a 6-degree cycle variation of horizontal spread angle of between 0.8° and 3.5°.

Figure 5a is an observation photograph indicating oriented states of the LC at the fringe region of the electrodes B and C, where electrode B is subject to a static state of m = 10 and V_B_ = 3.2–40.0 V (distributed symmetrically about the x-axis) with t_B_ = 1.0 ms. As seen in Fig. 5a, some disclinations are seen at the middle voltage regions, resembling soap bubbles, along the etched lines of the ITO layer (see Supplementary information S5 and S9).

Figure 5b shows the relationship between the amount of light radiated from the coupler C after passing through the region of electrode B and the constant voltage V_B_ applied to the electrodes B_k_ with the cycle t_B_ of triangular AC voltage as the parameter. When the cycle t_B_ was made longer than 2 ms, the observed horizontal spread angle of the radiated light increased gradually, expanding infinitely at over 10 ms. Whereas, when the cycle t_B_ was reduced to the range of 1/4–2.0 ms, while the spread angle became smaller, the amount of the radiated light declined and disappeared up to 0.1 ms.

Thus, the cycle conditions were set here as t_B_ = 1 ms, and the periodic number is confined to m = 10, instead of m = 5, to increase a deflective power generated by the electrode B (see Supplementary information S10).

Vertical sweeping is caused by the change in ERI for the coupler C based on the formula of Eq. 1. Figure 5c1–c3 show observation photographs of vertically sweeping beams radiated from the coupler C (corresponding to the area just visible in Fig. 5a) using the imaging camera of the f-θ lens (see supplementary movie M2). Their spots are generated by vibrating control of t_C_ = 1/32 ms and V_C_ = 0–20.0 V for electrode C.

The amount of light rises and falls between half of a sweep cycle: this can be explained by using the result of Fig. 3f, where the states of Fig. 5c1–c3 correspond to n_L_ ≥ 1.65, ≈ 1.62, and ≤ 1.59 for the curve of λ = 0.94 µm, respectively. In Fig. 5c1,c3, the guided light passes through the region of coupler C before being totally radiated because the fabricated groove depth (0.013 µm) was smaller than the designed value (0.02 µm). The horizontal collimation is slightly changed with changes in the radiation angle, but it can be compensated by controlling the envelope shapes of the signals applied to B_k_ according to the voltage of C.

Figure 5d,e show the vertical-steering performance for the voltage V_C_ applied to the electrode C with the cycle t_C_ of rectangular AC voltage as a parameter. They are measured under the same area shown in Fig. 5a. In Fig. 5d, the vertical displacement (or the sensitivity of vertical motion) peaks in the range of t_C_ = 1/8–1/32 ms and becomes about 10 degrees for the applied voltage of 20 V. (The displacement for t_C_ = 1 ms is half of that for t_C_ = 1/8–1/32 ms. Similarly, the maximum value of the refractive effect produced by the B_k_ electrodes for t_B_ = 1 ms will be also reduced by half. This is the reason that m = 10 was selected in this experiment.) As shown in Fig. 5e, the horizontal spread angles (FWHM of spread angle) are stable within 0.3° in the range equal to or less than 1/16 ms, and the vertical ones are stable within 0.6°–0.8° for any cycle range.

Figure 5f shows the frequency response of the vertical displacement. We can see that the half maximum of the displacement amplitude is maintained up to 100 Hz.

Basically, a frequency response of LC for electrode B has the same as that for electrode C which is shown in Fig. 5d. The reason why the results of Fig. 5b show a faster damping for applied voltage and AC frequency, is affection by the disclinations shown in Fig. 5a. Although measuring the frequency response of the electrode C can be protected from affection by disclinations (i.e., by using lower frequency and lower voltage of the electrode B), measuring that of the electrode B is directly affected because guided light definitely passes through many points where disclinations are generated. The guided light is perturbed irregularly around the disclination line, and this is thought to be the cause that the light decays with higher frequency and higher voltage as shown in Fig. 5b.

Figure 5g shows rotational sweeps in the rotational state of T_B_ = 0.5 s for electrode B and the static state of t_C_ = 1/16 ms and V_C_ = 15.0 V for electrode C (see Supplementary information S7and movie M3).

Figure 5h shows image-analyzed results of a movie according to Fig. 5g. The amount of light is measured while stationary along the vertical line (according to argument φ = 0° of Fig. 5g or the x-axis direction of Fig. 5a) of 6 pixels’ width from each image. The amount of light rises towards a peak over a cycle of 36°. The variation of the peaks is due to the shortage of sampling numbers, which is a function of the frame rate of the image sensor. Figure 5i shows relationships between the spread angle and the rotating angle φ corresponding to the spots shown in Fig. 5g. As shown in Fig. 5i, vertical spreads settle within 0.2°–0.3° and horizontal spreads vary over a 6-degree cycle (i.e., the divided angle of electrode B_k_) at 0.8°–3.5°. This cycle change is caused by a deficit of phase correction for a single beam, because only three electrode lines per single beam can contribute to beam-collection as shown in Fig. 5a. The change could therefore be improved by, after overcoming problems such as disclinations, changing the periodic number from m = 10 to m = 5 or increasing the division number of electrode B.

## Conclusion

We have demonstrated the world’s first two-axis scanner using liquid crystal control in which the scanning ranges were 360 degrees in the horizontal direction and 10 degrees in the vertical direction: the beam of a spread angle of 0.3° × 0.8° at minimum sweeps vertically up to 100 Hz frequency and ten equally-spaced beams scan rotationally with a 6-degree cycle variation of spread angle of between 0.8° and 3.5°.

The performances of spread angles and motion ranges can be improved by dealing with challenges like the disclinations at high voltages. The sweep frequency can be also improved by changing the thickness of the LC layer from 5 µm to less than 0.5 µm (see Supplementary information S11), since our device exploits the change at the surface layer interface of the LC. Because the frequency response of the LC is inversely proportional to the square of the thickness, a sweep frequency of more than 10 kHz (or 50 kHz if we add the five-beams effect) can be anticipated.

The input efficiency of coupler A for incident light, which is circularly polarized, falls to 40–50% because only TE mode light can be excited, and this coupled light can be fully radiated only from coupler C. The input efficiency of coupler C for return light, which is randomly polarized, also falls to 40–50% because only TE mode light can be excited. In the experiment, these efficiencies were thought not to be sufficient because the groove depth was shallower than the designed value. If a PBS and a half mirror are added to the system, outward efficiency will decrease by half (i.e., to 20–25%) and return efficiency will fall by 3/4 (to 30–37.5%).

Although production errors in as refractive indexes, wavelength, and layer thicknesses, etc., affect input coupling at coupler A and radiation at coupler C, these effects are to some extent counteracted by the LC control (see Supplementary information S12).

Some mechanical LIDARs originally have a 360-degree scanning range in the horizontal direction and their detection structures are used concomitantly with the emission structures. However, all conventional SS-LIDARs significantly narrow their ranges of motion to enable non-mechanical scanning, and most of them (except for MEMS-LIDARs) require detection structures that are separate from their emission structures.

Our CGC-LIDAR maintains a 360-degree scanning range and the detection and emission structures can be combined. Moreover, it produces multi-sweep beams that provide a higher-definition image enabling the “level 5,” and the S/N ratio of TOF signals detected using our method is anticipated to be 50-fold that achieved by conventional methods using a band pass filter due to the effect of the wavelength selectivity of the coupler (see Supplementary information S13).

We believe our method closely matches the requirements of SS-LIDAR. We will aim in future to achieve better performance and will at the same time investigate the potential for a low-cost mass-fabrication process.

Liquid crystal technology has seen considerable success in many areas. We anticipate that our experiments in LIDAR will open a new and expanding field for its use.

## Materials and methods

### Construction materials

The rod lens and CP plate are made of S-BSM14 by Ohara. The HR plate is made of BOC30 by Sumita. For the liquid crystal, 5CB (4-cyano-4'-pentylbiphenyl by TCI) is used.

### Measuring equipment

Light emitted from a DFB-LD (EYP-DFB-0935 by Eagleyard) is collimated and shaped into a parallel beam with circular spread by a collimator (C230TMD-B mounted on LDH3-P1/M by Thorlabs) and a beam expander (anamorphic prism pairs #47-274 by Edmund). After a portion of light is split by a half mirror (Non-Polarizing Beam splitter CCM5-BS017/M by Thorlabs) and coupled by a fiber coupler (fiber port PAF2P-15B by Thorlabs), the light’s wavelength is monitored by a light spectrum analyzer. The beam transmitted by the half mirror is adjusted by rotation of a λ/2 plate to minimize transmission through a polarization beam splitter (PBS, CCM5-PBS202 by Thorlabs). The light reflected from the PBS is circularly polarized by a λ/4 plate and is vertically focused to a CGCP by a focal lens (f = 100 mm). Since a portion of the incident light is reflected from coupler A and the half mirror (BS011 by Thorlabs), the focused spot is observed with the RLE and the CGCP using a microscope camera (DINOAM7915MZTL by Dino). The CGCP is located on a Peltier device (VPE20-30S by VICS) and is thermally controlled at 26 °C. The Peltier device is also located on the Gonio-stages and x- and y-stages for positioning. Light radiated from coupler C is converted by an f–θ lens (LSM05-BB by Thorlabs), collimated by a relay lens (LB1723-B by Thorlabs) and observed by an imaging camera that includes an image sensor (CM3-U3-13Y3M-CS by FLIR). The f-θ lens unit is set up on a vertically rotating mechanism. Voltage signals applied to the electrodes of the CGCP are produced in waveform generators (AWG-10 by Elmos) controlled by a computer and are magnified by up to fourfold by an amplifier.

### Estimation of spread angles

Spread angle is estimated by calculating the standard deviation of an intensity distribution along the vertical or horizontal cross-section passing through a peak point driven from jpeg images. The intensity is expressed by an integer value from 0 to 255: some of the lower range can be cut off as noise. Since the cutoff level affects the calculated result of the spread angle, it must be carefully identified and selected. The level is set here at 3% (the level for a black background) of 255.

## Supplementary Information

Supplementary Video 1.

Supplementary Video 2.

Supplementary Video 3.

Supplementary Information 1.
