# Supplementary material for: Biaxial 360-degree scanning LIDAR using a liquid crystal control
Source: Sci Rep. 2021 Jul 20;11:14767. doi: 10.1038/s41598-021-94208-2 (PMC8292532; doi:10.1038/s41598-021-94208-2)
Supplement: Supplementary file 4 — Supplementary Information 1. [file 41598_2021_94208_MOESM4_ESM.pdf]

Supplementary Information for

Biaxial 360-degree scanning LIDAR using a liquid crystal control

Seiji Nishiwaki

S1. Separation method of TOF signals

Diffused light reflected from an external object returns to the coupler C in the opposite direction. Although the direction of polarization of the return light is random, that of the coupled light is aligned in the rotational direction because only TE mode light is excited and guided. The polarization of light output from the coupler A is identical to that of the guided light. Figure S1a shows the relationships between the return beams (output from the coupler A) and their directions of polarization. The directions of polarization are orthogonal to the azimuth directions of the return beams. The five equally spaced beams (D_1 to D_5) return to photodiodes A and B with time lags in proportion to their distances from external objects: they are detected by photodiodes A and B and produce five TOF signals. It is therefore necessary to separate these five signals to be able to identify their mutual differences. While detection by photodiode A is independent of polarization, that by photodiode B is dependent on polarization due to having passed through the λ/4 plate and the PBS. Therefore, when the transmission axis of the PBS is aligned along the x-axis, the relationships between the angle φ and ratio P_b / P_a for the beams D_m are expressed by the equations of $P_b/P_a = \sin^2\{\varphi - 2(m-1)\pi/5\}\ for\ m = 1\ to\ 5$, as shown in Figure S1b, where φ is the azimuth angle of the beam B_1, and P_a and P_b are the values detected by photodiodes A and B, respectively. Since the five curves of P_b / P_a have different levels at an appropriate value φ, the five beams are separable based on the differences in their P_b / P_a ratio. Discrimination may be difficult at the crossing points of the curves, but it can be improved by comparing signals with a short time difference or by generating intermittent beams by controlling the applied voltages.

S2. RLE and vertical sweep by the applied voltage of the electrode C

Figure S2a shows a cross-sectional diagram of the RLE (rod lens element), on which ray-diagrams of light radiated from the coupler C for n_L = 1.53, 1.65, and 1.70 are superposed. Figure S2b shows a combination of a perspective illustration of a return beam reflected from a conical reflector and a cross-sectional drawing of return light refracted at the rod lens surface. The rod lens (refractive index 1.58) is 1.9 mm in diameter and 5 mm in height. Its upper, lower, and side surfaces are polished. The CP plate (refractive index 1.58), with a polished lower surface, is 20 mm in diameter and 2.3 mm in height, and a polished hole, shaped as a truncated cone with a base angle of 77 degrees and a base diameter of 3.4 mm, is formed at its center. If the expected change (from 1.53 to 1.71) of the refractive index of the LC can act on the guided light, vertical angular variation of more than 25 degrees can be anticipated.

Because radiated light scans rotationally on a conical surface, it is necessary to

convert the scanning surface to a horizontal plane. This can be achieved by setting up a reflector with a conical surface that has a large radius R. Since the beam spreads slightly in the horizontal plane due to reflection from the conical reflector, it needs compensating collimation control by the applied voltage of the electrode B according to the value of R. On the other hand, since the radius of the reflector is different within the beam diameter, after a return parallel beam reflects from the conical reflector and passes through the rod lens, it is focused on a focal line that is slightly inclined to the center axis L of the rod lens so as to make it broaden towards the top as shown in Fig. S2b. The conical reflector can therefore also help to correct the aberration caused by a declination of the focal line L_1 where light radiated from coupler C is focused.

S3. Grating patterns at the areas of B and C

Figures S3a and S3b shows top-view SEM photographs of coupler B and C. Concentric circular gratings (d = 0.013 μm deep) are formed concentrically at the areas of B and C (radius 0.05 - 4.50 mm and 4.50 - 6.50 mm) along the axis L by electron lithography on the SiO_2 layer after a planarizing process using CMP. While the groove duty rates for A and B are 0.5, that for C is 0.25.

S4. Electrode pattern

Figure S4 shows a microscopic photograph of areas A and B_k of an ITO layer. Electrode B is divided into 60 areas of B_k, which have a zigzag shape from the inner side to the outer side.

S5. Direction of alignment of LC molecules

In our device, the optical axes of LC molecules are oriented along the grating direction at the upper and lower boundary surfaces. All the directions are homogeneously aligned in the planar and thickness directions, as shown in Figs. S5a and S5g. When AC voltages are applied to the upper and lower electrodes, the directions tilt at an angle θ_T in response to the electric field intensity in the plane parallel to both the thickness direction and the grating direction, as shown in Figs. S5b, c, and h. The angle θ_T increases with rising AC voltage. This tilt causes the refractive index to change in the evanescent field of TE mode guided light. Since the grating orientation does not generate an LC pretilt, unlike with rubbing methods, the molecule direction at the boundaries is parallel to the surface. It therefore has two possible directions in which to turn when AC voltages are applied, as shown in Figs. S5h and S5i. If the upper and lower grating surfaces have no surface irregularities,

either direction is chosen and its pattern extends across the entire area according to the LC's alignment attribute, like one domino falling after another. The tilt angle of the molecules near the boundary continuously increases from zero to θ_T within thickness d_T. While the angle θ_T is dictated by the amplitude of the AC voltage, the thickness d_T is determined by the frequency of the AC voltage. Since the thickness d_T is very small (sufficiently less than the wavelength) in comparison with the LC thickness, its effect can be ignored in general-use LCDs. However, it has a major effect in our device, which uses the ERI changes in guided light, and the AC frequency significantly affects the performance of our device.

If the grating surface has a deep difference line, it disturbs a spread of the LC's alignment attribute, and in some case another tilt direction may be generated from the line, as shown in Figs. S5d - f. The directions of the molecule in Figs. S5d and S5f are close to being in the horizontal and vertical directions, as are those of Figs. S5a and S5c: they correspond to the conditions of the lower and higher voltages in Figure 5a. However, in Fig. S5e, the difference between LC orientations is obvious. This state corresponds to the condition of the middle voltage of Fig. 5a, i.e., "disclination," and its boundary is called a disclination line. LC alignment is thought to be perturbed irregularly around the disclination line, with the normal flow of the guided light being prevented. This is thought to be the reason for the light decaying with higher frequency and higher voltage as shown in Figure 5b.

S6. Horizontal collimation by applied voltage at electrode B

In Figure S6, guided light rays are drawn in red and radiated light rays are drawn in blue. Figures S6a and S6b show a ray trace where the optimally controlling voltages are applied to the electrodes B_k between 0 - 36 degrees and 0 - 360 degrees, respectively. As shown in Fig. S6b, five collimated beams are radiated equiangularly from the rod lens. Figure S6c shows a ray trace where the controlling voltages are shut off, and the radiated light is not collimated and instead diffuses straight from the rod lens in the same way as shown by the blue arrows in Figure 2d. Figure S6d shows a ray trace with the rod lens removed while applying the controlling voltages. It is also not collimated and is generally identical to the result shown in S6c. The radiated light is condensed to a parallel beam only by inserting the rod lens.

S7. Signals applied to electrodes B and C

Figures S7a, S7b and S7c are the signals applied to B_0, B_1, and C, where the equations of the envelope waveforms to time-axis are shown using the variable v as the applied voltage and the variable t as time. Figures S7d and S7e are extended

versions of Figs. S7a and S7c, in which the waveforms are formed by the triangular AC signal of the t_B cycle and the rectangular AC signal of the t_C cycle. Figures S7a, S7b, and S7d depict the signals applied to the B_k electrodes in Fig. 5g, that is, $T_B = 0.5$ sec, $2v_{mx} = 30$ volts, $2v_{mn} = 3.2$ volts, and $t_B = 1.0$ ms, where v_{mx} and v_{mn} are the maximum and minimum values of the respective waveform amplitudes. Figures S7c and S7e correspond to those applied to the electrodes C in Figs. 5c1 – 5c3, that is, $T_C = 1$ sec, $2v_{mx} = 15$ volts, $2v_{mn} = 0$ volts, and $t_C = 1/16$ ms. The phase difference δ between the signals applied to the neighboring electrodes is $T_B/6$ as shown in Figure S7b. Rotation of light radiated from coupler C is caused by the time-variable signal amplitudes and this phase difference.

It should be noted that there were upper and lower limits to both the sampling numbers and synchronization errors of the waveform generators used (AWG-10 by Elmos), i.e., 262144 points and ± 50 ppm (frequency deviation of the crystal clock). Opposite AC voltages are synchronously applied to the upper and lower electrodes to double the voltage difference. As shown in Fig. S7a, a triangular shape that was expressed by even step differences was employed for electrode B because it was resistant to synchronization errors due to having the smallest step difference. With electrode C, the synchronization errors cannot be ignored because the frequency of AC voltages exceeds those of electrodes B. The upper electrode (ITO electrode) was therefore connected to the ground and only the lower electrode (Al electrode) was used. With Fig. S7c, to produce the long waveform seen in Fig. S7c, a rectangular shape was employed that could express a waveform at the lowest points and which was higher in effective voltage than the triangular shape.

S8. Aberration correction for a parallel beam

As shown in Fig. S8a, if light radiated from the coupler C has a cylindrical wavefront and focuses onto a focal line L_l, the light after passing through L_l does not become a parallel beam after refraction by the rod lens. To make the beam wavefront flat, as shown in Fig. S8b, the radiated light must have a given aberration (i.e., a corrective aberration) which is related to the curve seen in Figure 2f. This aberration is discretely reproduced by the voltage shape applied to electrodes B as shown in Fig. S7a, but its accuracy is limited by an index formula that divides the division number (= 60) of electrodes B by the periodic number m. When m = 5, from ray-trace simulation, the spread angle of the parallel beam becomes less than 0.3 or 0.1 degrees at a standard deviation for the division number of 60 or 120, respectively.

S9. Surface roughness of the ITO layer

On examination of the CGCP sample, some disclinations were observed in the LC layer. This is thought to be caused by the etching difference of the ITO layer due to isolation of electrodes or wire lines and also by the fact that the surface of the ITO layer had picked up the surface roughness of the HR plate that had resulted from the acid cleaning process. Figure S9a shows a top-view SEM photograph of the surface of the HR plate after forming the ITO layer. The etching difference (100 nm) and a grain size of about 0.1 μm are observed here. The surface roughness of the ITO layer, Ra = 7 nm, is thought to result from the acid cleaning process of the HR plate. Figure S9b shows a cross-sectional SEM photograph of the surface of the HR plate before formation of the ITO layer (i.e., the grating surface of the SiN layer). Roughness of the order of several nanometers remains.

S10. Periodic number m and deflective power

Figures S10a and S10b depict the signals applied to the B_k electrodes for m = 5 and m = 10. Their period widths correspond to 72 and 36 degrees, respectively. The signal envelope of Figure S10b has a greater curvature than that of Figure S10a because they have the same amplitude. Figures S10c and S10d depict ray-traced results of guided light corresponding to Figs. S10a and S10b, respectively. Due to the deflection caused by the applied voltages, the wavefronts of guided light deviate from those for off-control. Figure S10d shows greater deflection power, and it reveals more variation of wavefront than in Figure S10c. Figures S10e and S10f depict respective equivalent models for Figures S10c and S10d to illustrate their difference. Light emitted from source O is focused by lens B of refractive index n_0 onto point F_0. The lens B in Fig. S10f has greater curvature than that in Figure S10e. If n_0 (corresponding to off-control) increases to $n_0+\Delta$, while the focal point F_0 of Fig. S10e moves to F_1' between F_0 and F_1, that of Fig. S10f moves to F_1 because Fig. S10f shows greater refraction power and reveals greater variation of wavefront than Figure S10e. This relation corresponds to that between Figs. S10c and S10d.

S11. LC thickness of 0.5 μm and its production challenges

While LCDs are fabricated with an LC thickness of greater than 2 μm, it is for optical reasons, not for process reasons. LCDs require a phase difference of more than λ/2 for visible light, so their thickness must be greater than 2 μm. Most LCD fabrication processes use sealing spacers made of an epoxy bonding agent incorporated with particulate silica to create two gapped glass panels. Our device can also use the same method. However, although LCDs need multiple in-plane spacers to maintain a uniform thickness, our device does not need any because it is smaller

than 20 mm in diameter. Since small-grain particulate silica is offered commercially (for example, ϕ500 ± 50 nm, 43-00-502 by Malvern), it is regarded as to posing fewer fabrication problem for our device. However, since it takes considerable time to inject LC into the small gap, the vacuum injection method may require some improvement.

S12. Production errors and temperature dependency of LC

Production errors in refractive indexes, wavelength, layer thicknesses, etc., affect both the input coupling at coupler A and radiation at coupler C. The effect on radiation changes the reference angle of radiation, but it can be to some extent counteracted by using electrode C to change the refractive index of the LC. The input coupling condition can be also compensated by LC control of electrode A. However, since the refractive index of LC is affected by temperature, thermal control using a Peltier device is necessary. When the temperature is raised from 26 to 32 ˚C, vertical angle θ (defined in Fig. S2a) passing through the rod lens increases by about 3˚. Figures S12a and b show the dynamic characteristics of displacement and horizontal spread angles of vertical steering beams under thermal control at 32 ˚C. These results are about the same as those shown in Figs. 5d and e (i.e., the results for thermal control at 26 ˚C).

S13. Wavelength selectivity and incident angle selectivity

A band pass filter (BPF) is configured by periodic layers such as the combination of an SiO_2 layer and a Ta_2O_5 layer as shown in Figure S13a. Due to the periodicity of the layer thicknesses, the BPF of thickness 8 μm can achieve a wavelength selectivity (or a band pass width) of 10 nm, as shown in Figure S13b.

Figures S13c and S13d show responses of wavelength selectivity and incident angle selectivity for coupler C to coupling length L, calculated by 2D-FDTD for Λ = 0.30 μm, d = 0.02 μm, ε = 0.2, t_1 = 0.14 μm, t_0 = 1.16 μm, and n_L = 1.65. The respective selectivity is defined as the variation in full-width of wavelength or incident angle that damps the peak input-coupling efficiency by half. When the coupling length of coupler C is L = 1.5 mm, the selectivity is estimated to be 0.2 nm or 0.03 degrees for wavelength or incident angle. The wavelength selectivity of 0.2 nm is about two percent of that of a common BPF. Thus, since our device is not affected by crosstalk signals from other LIDARs, it is a suitable property for LIDAR. Additionally, the selectivity of the BPF is about the same as that obtained at a coupling length of w = 8 μm on the curve in Figure S13c. This is because both interference and coupling follow the phase matching principle.

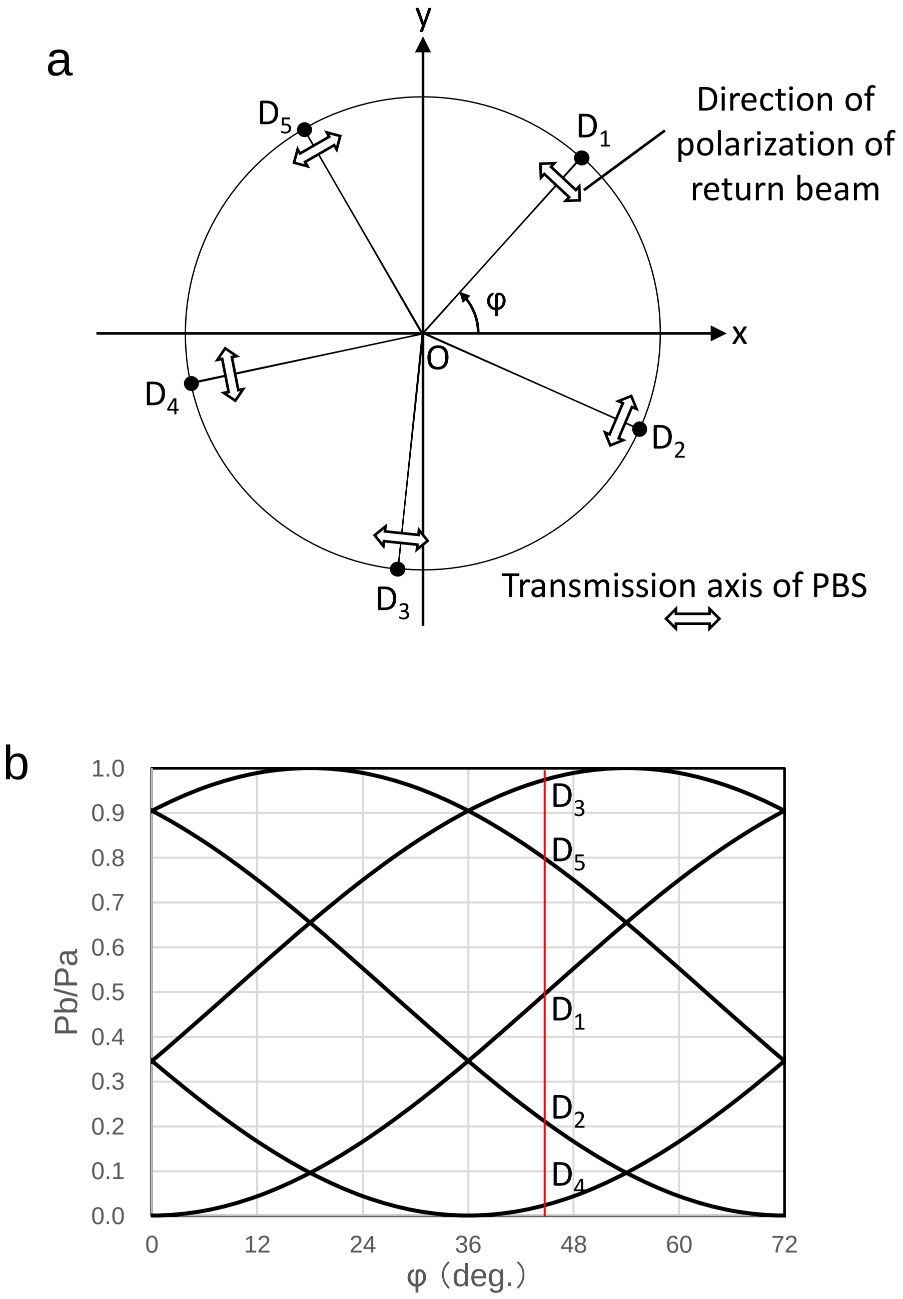

Figure S1. Separation method of TOF signals

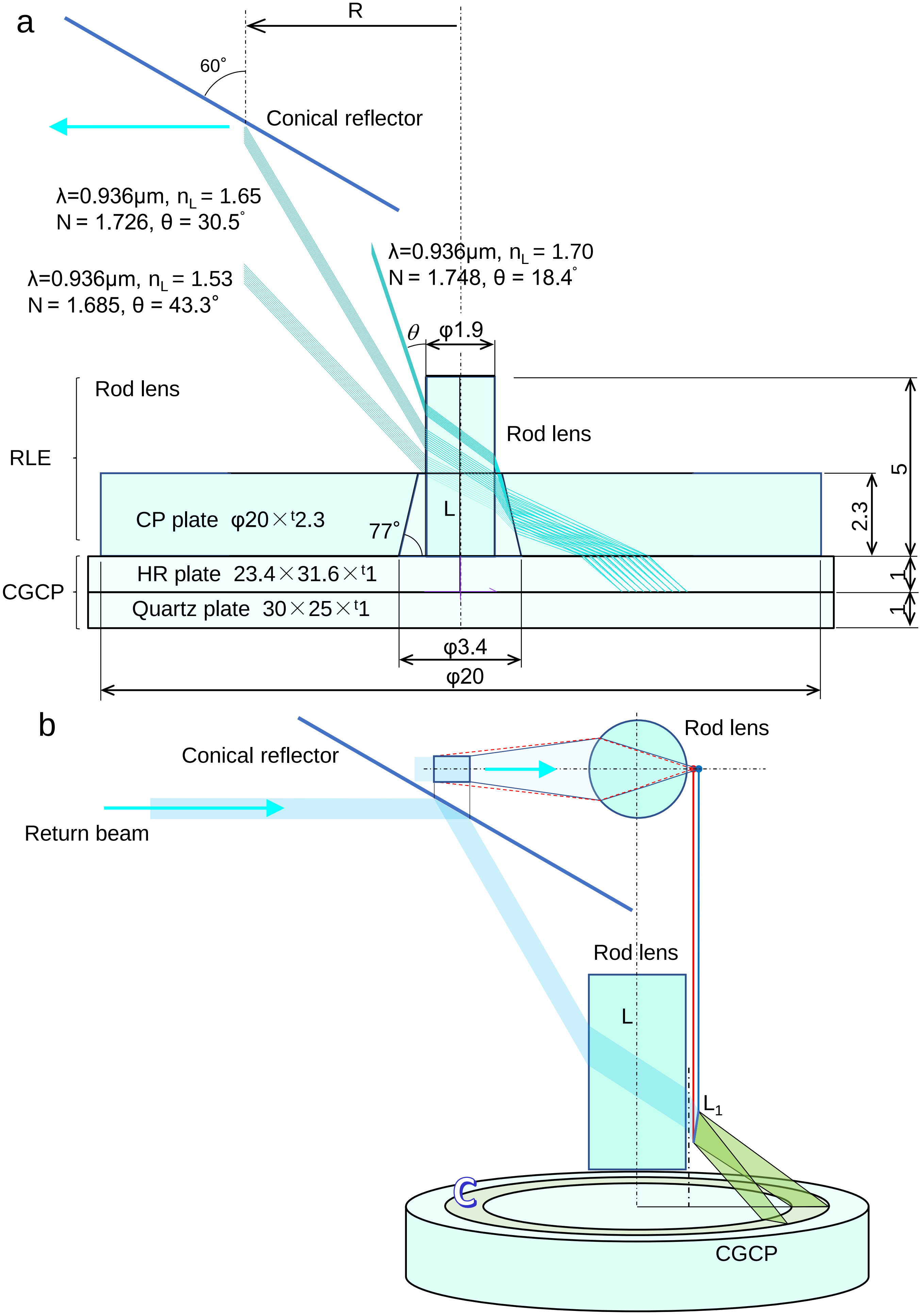

Figure S2. RLE and conical reflector

a b

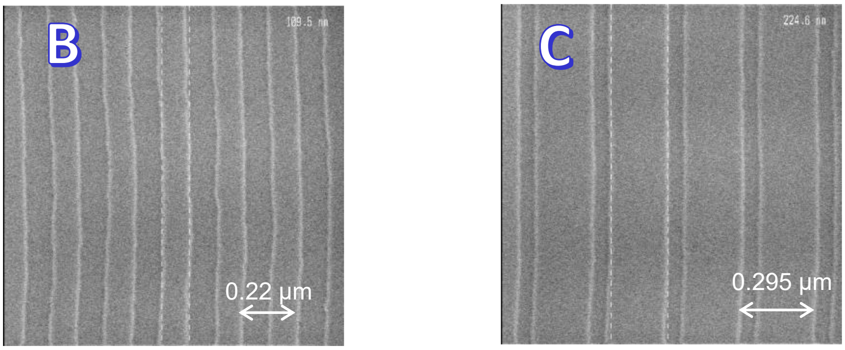

Figure S3. SEM photographs of the Ta_2O_5 surface

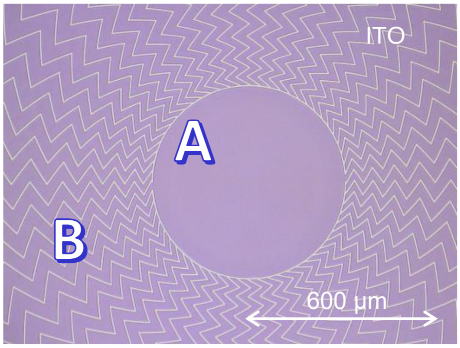

Figure S4. Microscopic photograph of the ITO electrode

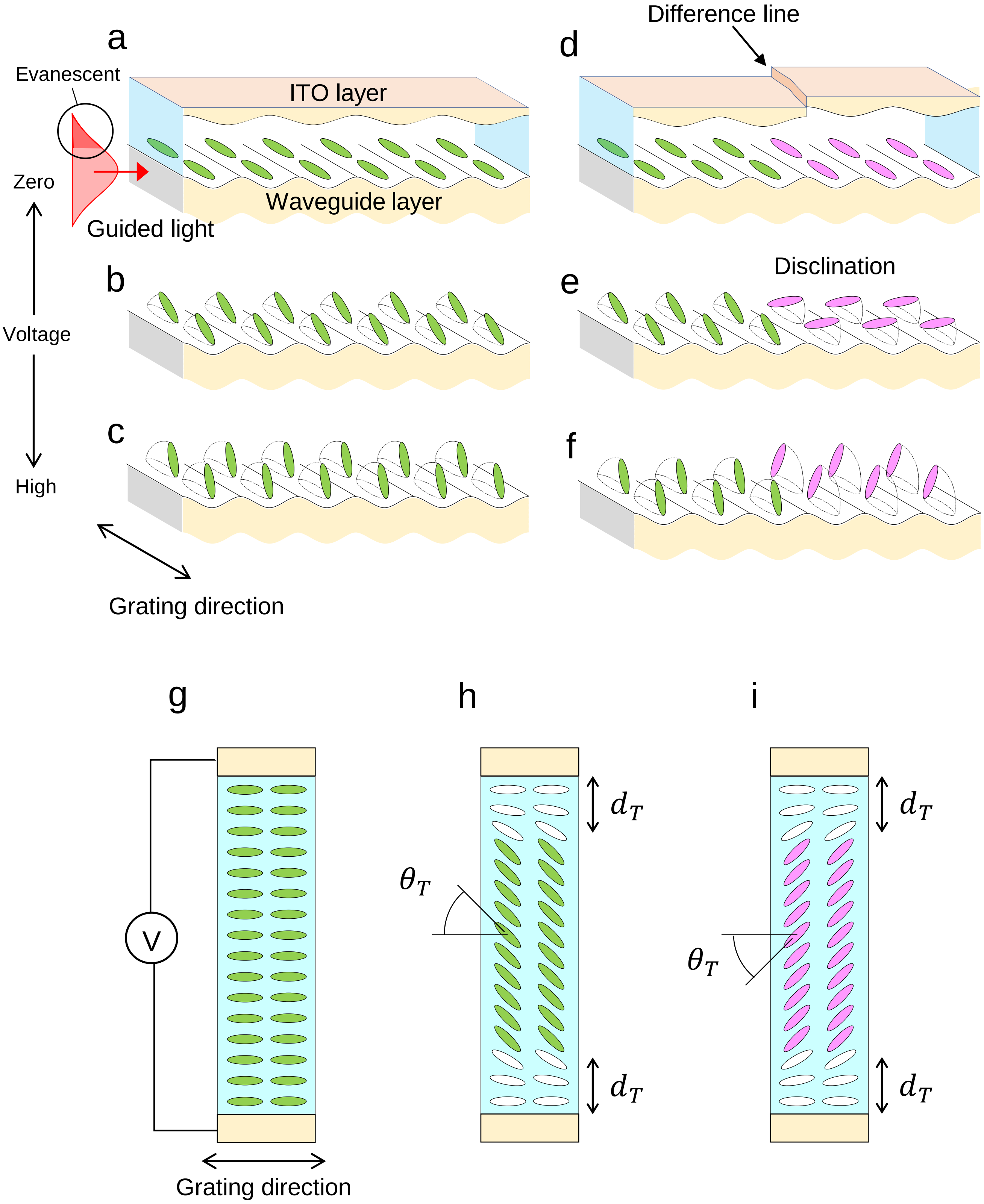

Figure S5. Direction of alignment of liquid crystal molecules

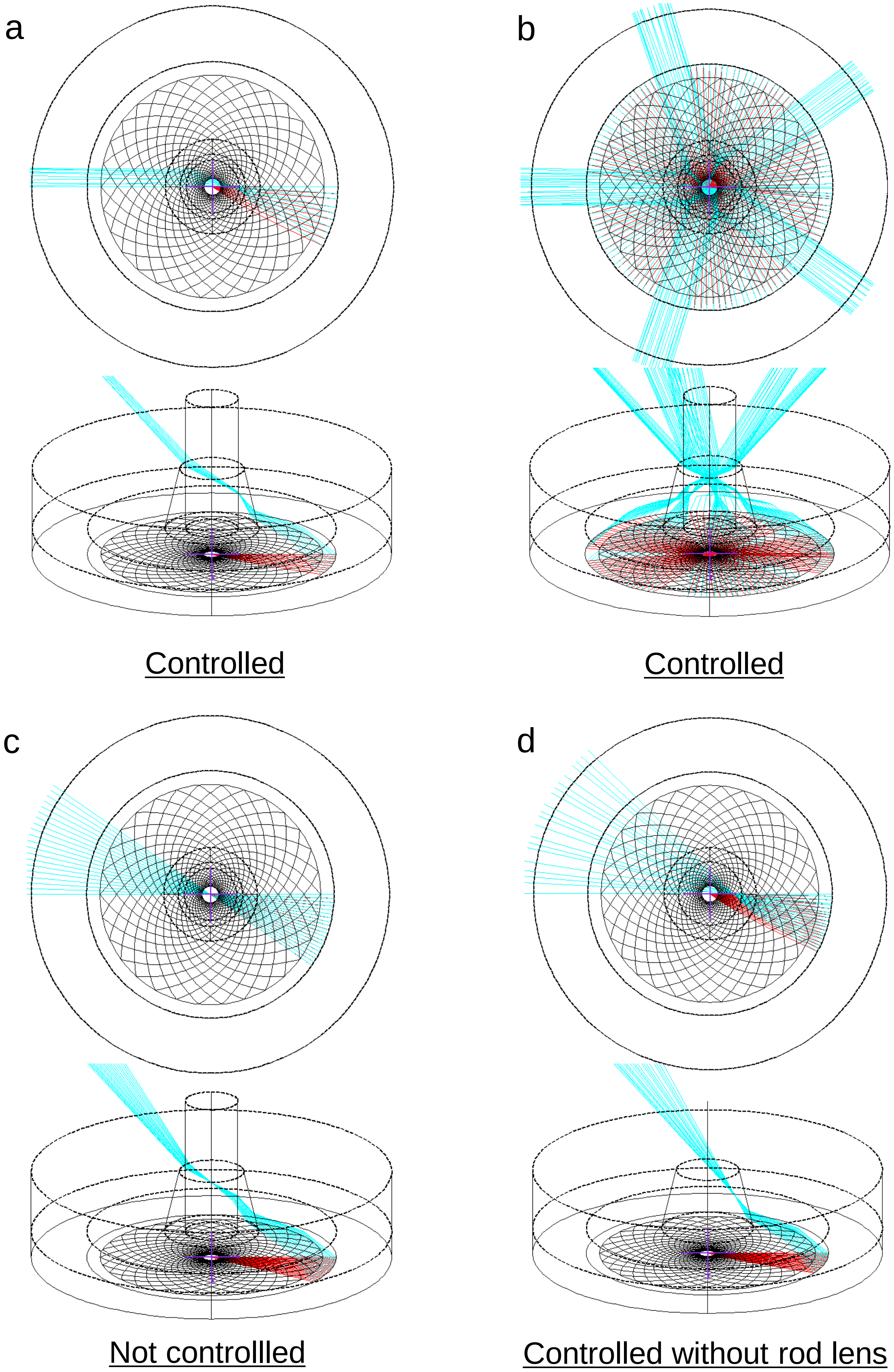

Figure S6. Ray traces for different conditions of LC control

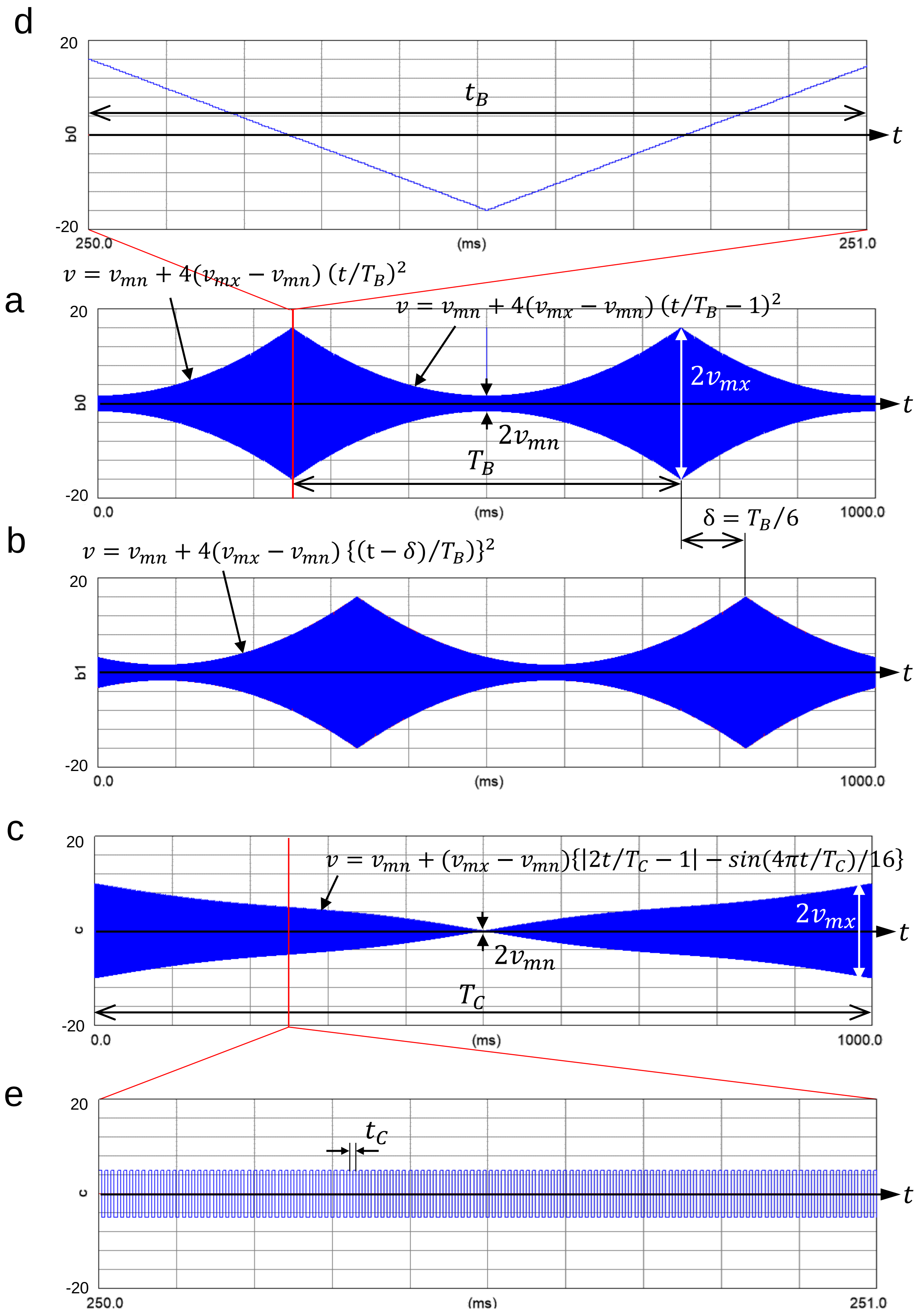

Figure S7. Signals applied to electrodes B and C

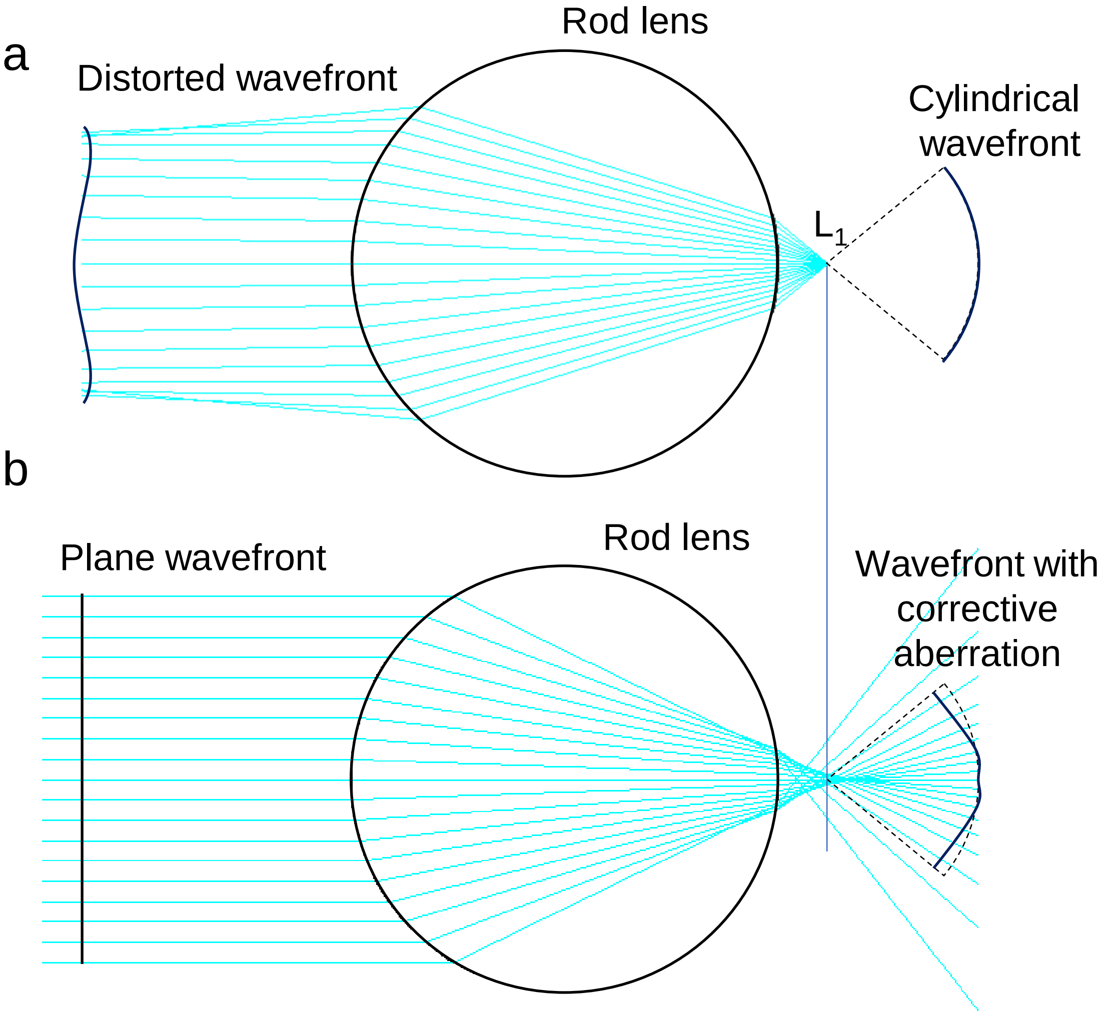

Figure S8.   Aberration correction for a parallel beam

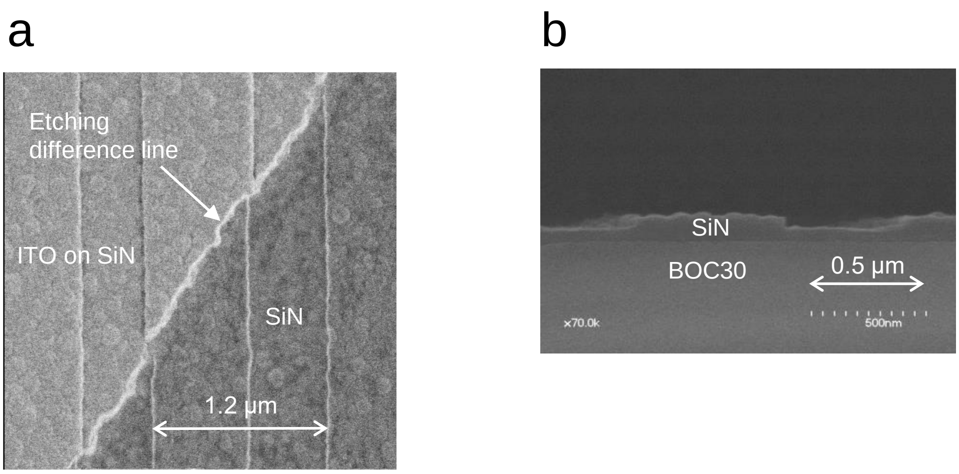

Figure S9.   Surface roughness of the ITO layer

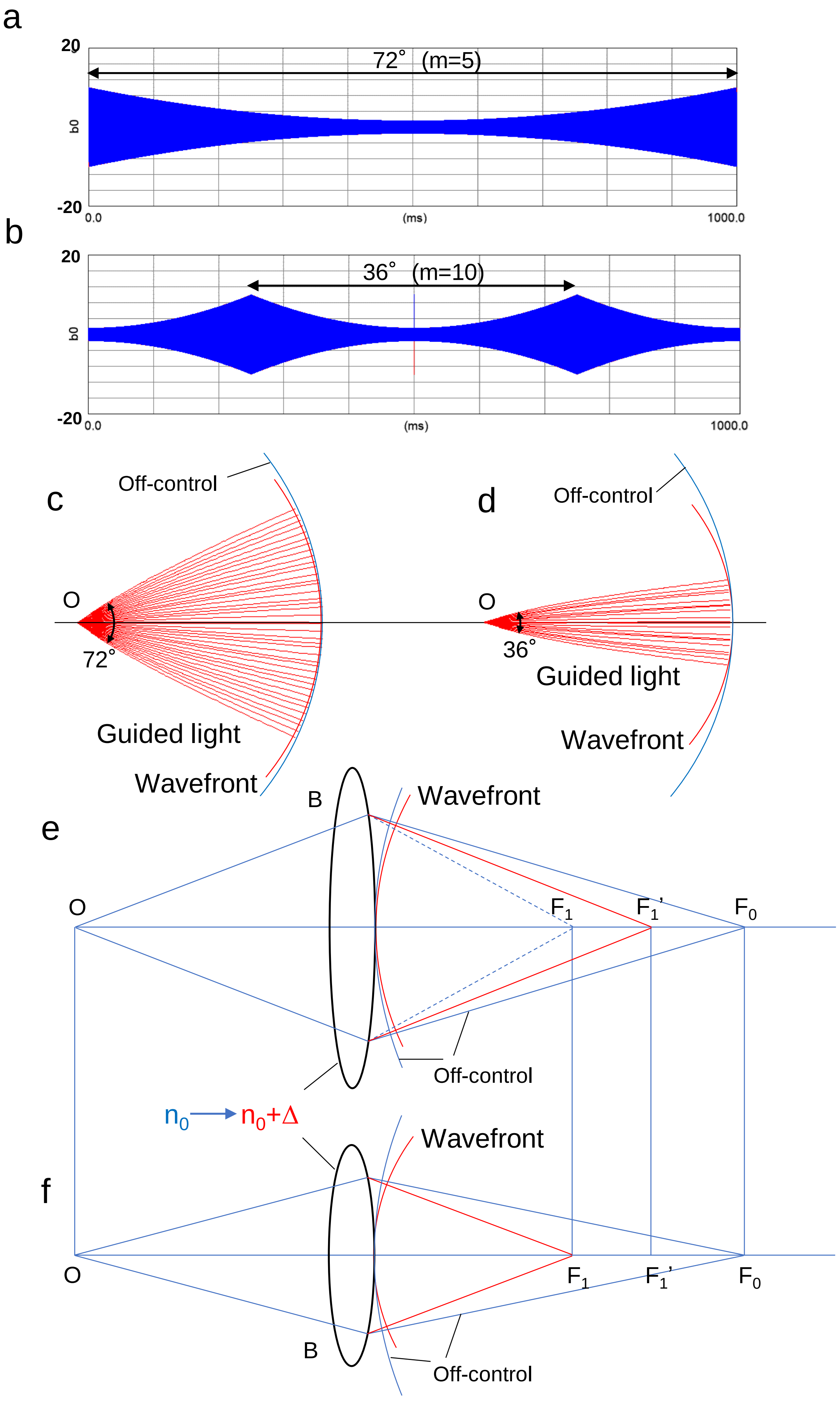

Figure S10. Periodic number m and deflective power

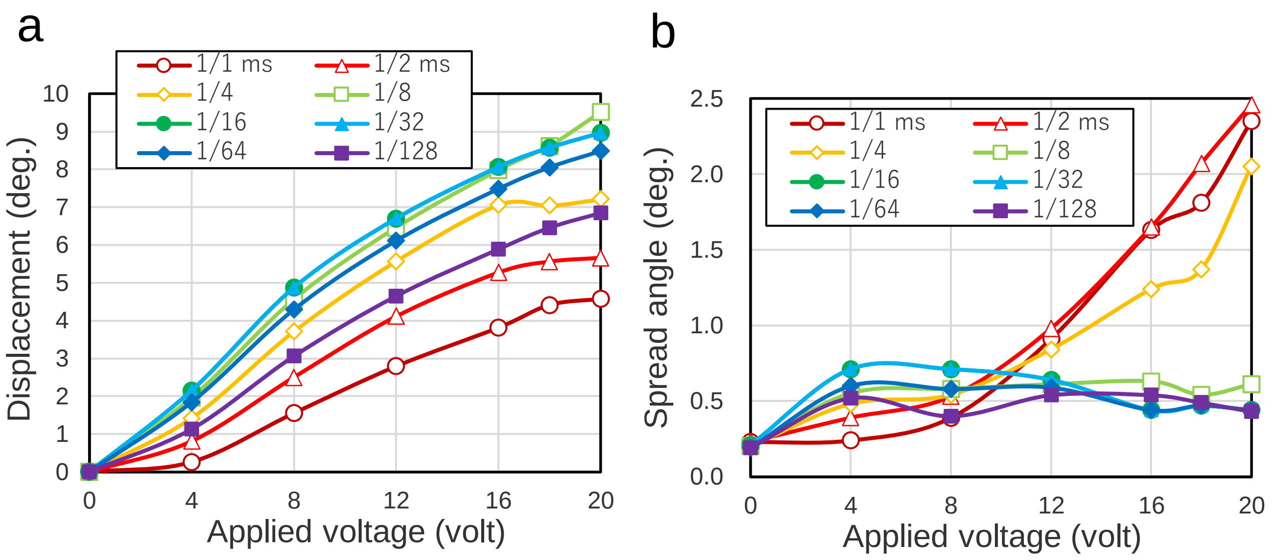

Figure S12. Temperature dependency of LC

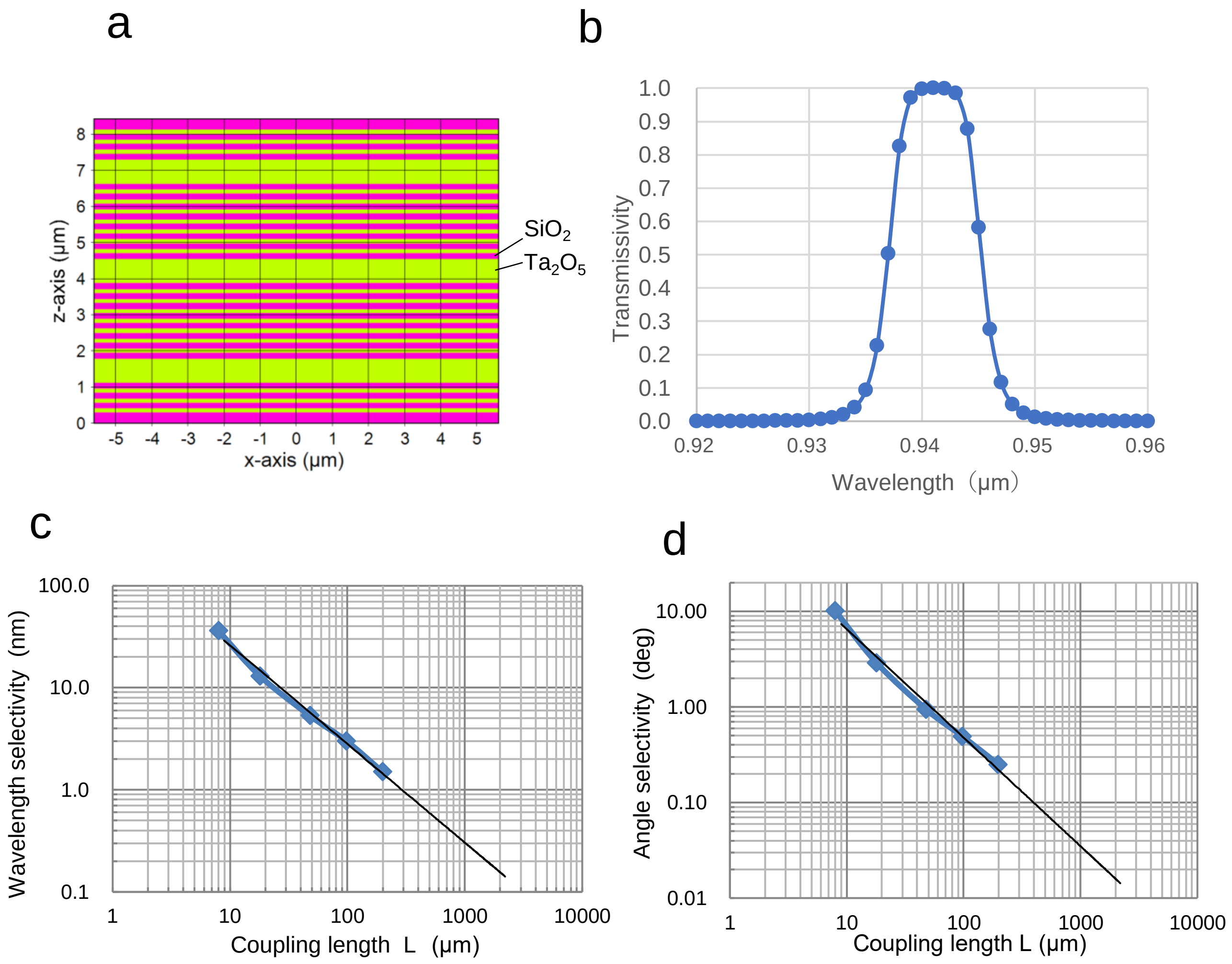

Figure S13. Wavelength selectivity and angle selectivity
